# Survival analysis for AdVerse events with VarYing follow-up times (SAVVY)—estimation of adverse event risks

**DOI:** 10.1186/s13063-021-05354-x

**Published:** 2021-06-29

**Authors:** Regina Stegherr, Claudia Schmoor, Jan Beyersmann, Kaspar Rufibach, Valentine Jehl, Andreas Brückner, Lewin Eisele, Thomas Künzel, Katrin Kupas, Frank Langer, Friedhelm Leverkus, Anja Loos, Christiane Norenberg, Florian Voss, Tim Friede

**Affiliations:** 1 Institute of Statistics, Ulm University, Ulm, Germany; 2 Clinical Trials Unit, Faculty of Medicine and Medical Center, University of Freiburg, Freiburg im Breisgau, Germany; 3 F. Hoffmann-La Roche, Basel, Switzerland; 4 Novartis Pharma AG, Basel, Switzerland; 5 Janssen-Cilag GmbH, Neuss, Germany; 6 Bristol-Myers-Squibb GmbH & Co. KGaA, München, Germany; 7 Lilly Deutschland GmbH, Bad Homburg, Germany; 8 Pfizer, Berlin, Germany; 9 Merck KGaA, Darmstadt, Germany; 10 Bayer AG, Wuppertal, Germany; 11 Boehringer Ingelheim Pharma GmbH & Co. KG, Ingelheim, Germany; 12 Department of Medical Statistics, University Medical Center Göttingen, Humboldtallee 32, Göttingen, 37073 Germany

**Keywords:** Aalen-Johansen estimator, Adverse events, Competing events, Drug safety, Incidence proportion, Incidence density, Kaplan-Meier estimator

## Abstract

**Background:**

The SAVVY project aims to improve the analyses of adverse events (AEs), whether prespecified or emerging, in clinical trials through the use of survival techniques appropriately dealing with varying follow-up times and competing events (CEs). Although statistical methodologies have advanced, in AE analyses, often the incidence proportion, the incidence density, or a non-parametric Kaplan-Meier estimator are used, which ignore either censoring or CEs. In an empirical study including randomized clinical trials from several sponsor organizations, these potential sources of bias are investigated. The main purpose is to compare the estimators that are typically used to quantify AE risk within trial arms to the non-parametric Aalen-Johansen estimator as the gold-standard for estimating cumulative AE probabilities. A follow-up paper will consider consequences when comparing safety between treatment groups.

**Methods:**

Estimators are compared with descriptive statistics, graphical displays, and a more formal assessment using a random effects meta-analysis. The influence of different factors on the size of deviations from the gold-standard is investigated in a meta-regression. Comparisons are conducted at the maximum follow-up time and at earlier evaluation times. CEs definition does not only include death before AE but also end of follow-up for AEs due to events related to the disease course or safety of the treatment.

**Results:**

Ten sponsor organizations provided 17 clinical trials including 186 types of investigated AEs. The one minus Kaplan-Meier estimator was on average about 1.2-fold larger than the Aalen-Johansen estimator and the probability transform of the incidence density ignoring CEs was even 2-fold larger. The average bias using the incidence proportion was less than 5%. Assuming constant hazards using incidence densities was hardly an issue provided that CEs were accounted for. The meta-regression showed that the bias depended mainly on the amount of censoring and on the amount of CEs.

**Conclusions:**

The choice of the estimator of the cumulative AE probability and the definition of CEs are crucial. We recommend using the Aalen-Johansen estimator with an appropriate definition of CEs whenever the risk for AEs is to be quantified and to change the guidelines accordingly.

## Background

Time-to-event or survival endpoints are common in clinical trials comparing different treatments in patients with a specific disease [1, 2], e.g., overall survival in oncological trials. The observation of the event times, such as the time to death, is typically incomplete, since not all patients experience the event of interest until the time of trial read-out. For some patients, it is only known that the event has not yet occurred during follow-up, and their time from trial entry to trial closure is called a censored observation. For the statistical analysis of this type of data, established survival analysis techniques are required, such as the well-known Kaplan-Meier estimator of the probability of being event-free over time. Sometimes, competing events (CE) have to be considered in addition. These are events that preclude the occurrence of the event of interest. As an example, in the ALEX trial investigators were interested in the secondary endpoint of “time to central nervous system (CNS) progression” [3]. A patient who experiences a non-CNS progression event cannot experience a CNS progression event later anymore, even if all patients would be followed up until their deaths. A patient who dies before progression cannot experience a later CNS progression event, either. So, the events “non-CNS progression” and “death” are CEs when considering the endpoint “time to CNS progression”. Standard survival analyses assume that in the long run every patient will experience the event of interest and will therefore give biased estimates in general. In the presence of CEs, dedicated statistical methods are required to give unbiased estimates, such as the Aalen-Johansen estimator (AJE) of the cumulative probability of the interesting event over time [4]. For the analysis of efficacy in clinical trials based on time-to-event endpoints in the presence of CEs, adequate statistical methods are well established, and a large amount of substantial literature exists on their adequate use [5, 6].

For the analysis of safety, the situation is different. In clinical trials, an essential part of the safety assessment of treatments is based on the analysis of adverse events (AEs). An AE is any unfavorable and unintended sign including an abnormal laboratory finding, symptom, or disease temporarily associated with the exposure to an investigational product, whether or not considered related to the product [7, 8]. AEs are documented by the clinical investigator and coded with the Medical Dictionary for Regulatory Activities (MedDRA), which provides clinically validated medical terminology (https://www.meddra.org/). In the analysis, interest focuses often on the risk of experiencing at least one AE of a specific type defined by severity or by MedDRA codes, as e.g., MedDRA preferred term or MedDRA system organ class or AEs of special interest in the indication under study. According to the European Commission’s guideline on summary of product characteristics (SmPC) [9, 10], the AE risk is classified into frequency categories which are defined by “very rare,” “ rare,” “uncommon,” “common,” and “very common” when the risk is <0.01%, <0.1%, <1%, <10%, and ≥ 10%, respectively. AEs can occur at any point in time during patients’ follow-up in a clinical trial. The follow-up times can be incomplete, leading to censoring, and can vary between patients and between treatment groups. Additionally to censoring, CEs can occur. The most obvious one is death without prior AE of the interesting type. So, the situation is not different from time-to-event endpoints for efficacy analyses in clinical trials. But statistical methods properly accounting for all these features of AE data are very rarely used in clinical trials. The analysis is usually much more simplistic and often ignores the time dynamic structure of AEs [8].

Estimation of the probability of an AE of a specific type within a specific time interval is often done by the simple incidence proportion, i.e., the number of patients with at least one observed AE of the specific type divided by group size. The worry is that the incidence proportion underestimates the cumulative AE probability because it does not account for censoring [4, 8, 11, 12]. Other proposals exist which account for censoring. One proposal is the (exposure adjusted) incidence density which divides the number of patients with at least one observed AE the by cumulative patient-time at risk. This does account for censoring but does not estimate a probability. It estimates the AE hazard assuming it to be constant over time. Under this rather strong assumption [13, 14] it may be transformed onto the probability scale [15].

A detailed methodological investigation of these concerns can be found for instance in [4]. The practical question for trialists is how to empirically quantify adverse event risk, which, in turn, also informs the AE frequency categories mentioned above. The Kaplan-Meier estimator has traditionally been used to quantify the empirical survival probability for the outcome all-causes death, taking into account patients for whom due to censoring at, e.g., trial closure, only a minimum survival time is known. Patients censored following trial closure are still alive and their time of death will remain unobserved. One minus Kaplan-Meier therefore is an approximation of the cumulative death proportions, and given sufficient follow-up data, it eventually approaches 100%.

The Kaplan-Meier method has also been used [16] or recommended (in the European Medicine’s Agency, EMA, anticancer guideline [17] or the extension of the CONSORT statement on reporting harms [18]) for outcomes such as AEs, now additionally censoring observed deaths without prior AE under consideration. The rationale is that prior death also prevents observation of the outcome of interest, but the approach ignores that, given sufficient follow-up data, the cumulative AE proportion will not approach 100% in the presence of such competing mortality. This is the conceptual reason why one minus the Kaplan-Meier estimator for AE outcomes is bound to overestimate the AE risk.

The incidence density operates on a different scale, taking patient-time rather than the number of patients as denominator, but a simple transformation (see the “Methods” section below) finds that it presents nothing but a parametric counterpart to the Kaplan-Meier estimator under a very restrictive parametric assumption [15]. In contrast, the incidence proportion operates on the same scale as Kaplan-Meier, but runs the risk of underestimation for the following reason. The incidence proportion could also be calculated for the outcome “observed all-causes death,” but—unlike Kaplan-Meier—would not be a proper approximation of the cumulative death proportions, because it is not able to include death events after censoring into the calculation. An analogous argument holds for AE outcomes.

The methodological literature therefore advocates the AJE as a generalization of the Kaplan-Meier estimator to multiple outcome types, because it is the corresponding nonparametric estimator that provides unbiased estimates in presence of varying follow-up times, censoring, and CEs. These multiple outcomes or CEs require defining what a CE is, including but not limited to death before AE. A detailed operationalization in the AE context is provided below. The AJE equals the AE incidence proportion in the absence of censoring and it equals one minus Kaplan-Meier in the absence of CEs. For all these substantive reasons, the AJE is the non-parametric gold-standard. There also is a parametric counterpart including a second incidence density for events such as death before AE [19].

The concerns above are qualitative. However, the amount of bias, comparing, e.g., the incidence proportion or one minus Kaplan-Meier with the non-parametric gold standard, the AJE [4] accounting for both CEs and censoring will depend on the specific trial setting. In particular, the relative frequencies of observed AEs, observed CEs and observed censorings add up to 100% at any point in time. The latter two are leading forces influencing bias, and, e.g., the presence of many CEs in a time-to-first-event analysis will impact the amount of censoring.

Here and for the trial data reported below, we are using the term “bias” with reference to the AJE, because AJE is an (asymptotically) unbiased estimator both in the presence of CEs and censoring, while at the same time not requiring a parametric assumption [20]. Hence, we are investigating an approximate bias with respect to the underlying true effect which remains unknown in real data analyses.

The SAVVY project group (Survival analysis for AdVerse events with Varying follow-up times) is a collaborative effort from academia and pharmaceutical industry with the aim to improve the analyses of AE data in clinical trials through the use of survival techniques that account for varying follow-up times, censoring, and CEs. Here, we report one-sample results from an empirical study of an opportunistic sample of randomized clinical trials from several sponsor companies. Our investigations are motivated by a typical trial setting and the (primary) safety patient set containing, in particular, the timing of adverse events. The data structure is characterized by varying follow-up times, CEs, and censoring as discussed above. This must be acknowledged in any analysis of AEs, be it for emerging AEs or prespecified AEs of interest. In addition, estimated AE risk also informs AE frequency categories. To illustrate, one reviewer pointed out that incidence proportions and incidence densities are typically seen for the analysis of unspecified emerging events, while Kaplan-Meier is more common for prespecified events of interest. However, above we have explained their connection and demonstrated the inappropriateness of incidence proportion, incidence density, and Kaplan-Meier methods in the presence of varying follow-up times, CEs, and censoring.

Hence, our aim is to illustrate the amount of empirical bias when quantifying absolute AE risk in single samples including categorization into AE frequency categories. Results when comparing safety between treatment groups will be communicated in a follow-up paper [21], building on the insights obtained in the present investigation.

## Methods

A detailed statistical analysis plan is available elsewhere [22]. Individual trial data analyses were run within the sponsor organizations using SAS and R software provided by the academic project group members. Only aggregated data necessary for meta-analyses were shared and meta-analyses were run centrally at the academic institutions. The meta-analysis is used for the methodological comparison. Its is a formal assessment of the bias including the variances of the estimates.

Here, we briefly summarize one-sample estimators and methods of meta-analysis. Properties and estimands of the estimators are discussed elsewhere [8, 22]. However, the conceptual rationale of the statistical analysis plan in conjunction with properties and estimands of the methods at hand have been presented in the “Background” section above. We describe in more detail the definition of CEs which has an immediate consequence on the estimation procedures.

AE probability estimators will be compared based on ratios taking the gold-standard AJE as denominator. The rationale for taking AJE as the gold-standard is explained below. Impact on frequency categorizations will be tabulated and the ratios of the estimators will be meta-analyzed.

### One-sample estimators

We will consider the following estimators of the cumulative AE probability or “AE risk” in a time-to-first-event analysis. Since both probabilities and the amount of censoring [23] are time-dependent, we will allow for different evaluation times called *τ*. These evaluation times either imposed no restriction, i.e., evaluated the estimators until the maximum follow-up time, or considered the minimum of quantiles of observed times in the two treatment groups; the quantiles were 100%, 90%, 60%, and 30%. We will report results from “Arm E,” denoting the experimental treatment groups. The incidence proportion is

1 $$ {}IP_{\mathrm{E}}(\tau) = \frac{\text{no. of patients w.\ observed AE on \([0, \tau]\) in E}}{n_{\mathrm{E}}},  $$

where *n*_E_ denotes sample size in group E. This estimator will be called *incidence proportion* in the following.

The AE incidence density is

2 $$ {}ID_{\mathrm{E}}(\tau) = \frac{\text{no. of patients w.\ observed AE on \([0, \tau]\) in E}}{\text{patient-time at risk in E restricted by \(\tau\)}}.  $$

Incidence densities are not directly comparable to, e.g., incidence proportions. A common transformation of the AE incidence density onto the probability scale is

3 $$ 1 - \exp\left(- ID_{\mathrm{E}}(\tau) \cdot \tau\right),  $$

called *probability transform incidence density ignoring CE* in the following. The *one minus Kaplan-Meier* estimator only codes observed AEs as an event and censors anything else on [0,*τ*]. It is defined by formula (4) in [22].

An incidence densities’ analysis accounting for CEs uses the competing incidence density

4 $$ \overline{ID}_{\mathrm{E}}(\tau) = \frac{\text{no. of patients w.\ observed CE on \([0, \tau]\) in E}}{\text{patient-time at risk in E restricted by \(\tau\)}}  $$

such that we get the following AE-probability estimator

5 $$ \frac{ID_{\mathrm{E}}(\tau)}{ID_{\mathrm{E}}(\tau) + \overline{ID}_{\mathrm E}(\tau)} \left(1-\exp(-\tau\cdot[ID_{\mathrm{E}}(\tau) + \overline{ID}_{\mathrm E}(\tau)])\right),  $$

called *probability transform incidence density accounting for CE* in the following. Finally, the *AJE* generalizes (5) to a fully non-parametric procedure and decomposes the usual one minus Kaplan-Meier estimator of the time-to-*any*-first-event (AE or competing) into estimators of the cumulative AE probability plus the cumulative CE probability [4]. It is defined by formula (8) in [22]. The AJE will serve as “gold-standard,” because it is asymptotically unbiased both in the presence of CEs and censoring, without the need to make a parametric assumption. As explained earlier and, in more detail, below, “bias” will be with respect to AJE and is the approximate bias as a consequence of the real trial data setting and the approximate unbiasedness of the gold-standard.

### Definition of competing events

The definition of events as “competing” is essential to both the AJE and the competing incidence density. CEs (or “competing risks”) are events that preclude the occurrence or recording of the AE under consideration in a time-to-first-event analysis. One important CE is death before AE. In addition, any event that would both be viewed from a patient perspective as an event of his/her course of disease or treatment and would stop the recording of the interesting AE will be viewed as a CE. To illustrate, premature discontinuation of study treatment which leads to end of AE recording will be handled as a CE [24]. Consequently, possibly disease- or safety-related loss to follow-up, withdrawal of consent and discontinuation is handled as a CE as this is typically related to an event associated with the disease course or therapy.

In order to investigate the impact of the definition of CEs, we also investigated a “death only” scenario, which only treated death before AE as competing, but not the other CEs. This estimator will be called *AJE (death only)* in the following.

### Aalen-Johansen as gold-standard

The data generation mechanism underlying the clinical trials is based on the hazard of the AE, the hazard of the CE, and the distribution of the censoring times, where the hazards are not restricted to be constant [22]. But not all estimators suggested for analyzing AEs can adequately deal with all three processes. Table 1 gives an overview whether the estimators account for the three sources of bias, i.e., censoring, no constant hazards, and CEs. The incidence proportion ignores CEs and censoring in the analysis in the same way as the respective patients are counted in the denominator as if they had been followed for the entire study period. This is a proper handling of the CEs as it correctly takes into account that an AE cannot occur after the patient had experienced a CE. It is an improper handling of censoring as it incorrectly implies that an AE could have been observed over the entire follow-up period, which is not true due to censoring.

**Table 1:** Overview whether the estimators deal with the possible sources of bias

	Accounts for censoring	Makes no constant hazard assumption	Accounts for CEs
Incidence proportion	No	Yes	Yes
Probability transform incidence density ignoring CEs	Yes	No (AE Hazard)	No
1-Kaplan-Meier	Yes	Yes	No
Probability transform incidence density accounting for CEs	Yes	No (AE and CE Hazard)	Yes
Death only AJE	Yes	Yes	Yes (Death only)
Gold-standard AJE	Yes	Yes	Yes

The AJE is the only estimator that is able to deal with all three potential sources of bias and is therefore considered the gold standard estimator and will serve as a benchmark for comparison of results. In the following, we will use the term bias for deviations of the estimators from this benchmark estimator and not for the difference to the true value. This is considered appropriate as the differences of the estimators to the AJE converge in probability to the asymptotic bias. Stegherr et al. [20] have more closely investigated this question, finding that investigating the “empirical” bias with respect to AJE well approximates the true bias with respect to the true quantity only known in simulations.

### AE frequency categories

According to the European Commission’s guideline on summary of product characteristics (SmPC) [9] and based on the recommendations of the CIOMS Working Groups III and V [10], the frequency categories of AE risk in the most representative exposure period are respectively classified as “very rare,” “rare,” “uncommon,” “common,” and “very common” when found to be <0.01%, <0.1%, <1%, <10%, and ≥ 10%. Frequency categories obtained with the different estimators will be compared to frequency categories obtained with the gold-standard AJE.

### Random effects meta-analysis and meta-regression

In the meta-analysis and meta-regression, the ratios of the AE probability estimates obtained with the different estimators divided by the AE probability obtained with the gold-standard AJE are considered on the log-scale. The standard errors of these log-ratios are calculated with a bootstrap to account for within trial dependencies. Then, a normal-normal hierarchical model is fitted and the exponential of the resulting estimate can be interpreted as the average ratio of the two estimators.

In a meta-regression, it is further investigated which variables impact this average ratio. Therefore, the proportion of censoring, the evaluation time point *τ*, i.e., the maximal time to event in years (AE, CE or censoring) observed under the given evaluation time, and the size of the AE probability estimated by the gold-standard AJE are included as covariates in a univariable and a multivariable meta-regression. The covariates are centered in the meta-regression.

## Results

### Description of the data

Ten organizations provided 17 trials including 186 types of AEs (median 8; interquartile range [3,9]). Twelve (71.6% out of 17) trials were from oncology, nine (52.9%) were actively controlled, and eight (47.1%) were placebo controlled. The trials included between 200 and 7171 patients (median 443; interquartile range [411,1134]). For the comparison of the AE probabilities, we focus on the experimental treatment group. The corresponding results of the control group will be reported in a follow-up paper on group comparisons [21]. Median follow-up of the treatment group was 927 days (interquartile range [449,1380]). In the experimental treatment group, the median of the calculated gold-standard AJE was 0.092 (minimum 0 and maximum 0.961). For one of the 17 trials, details of the trial and the AE analysis by the different methods investigated in this paper are presented in [20].

**Fig. 1:**
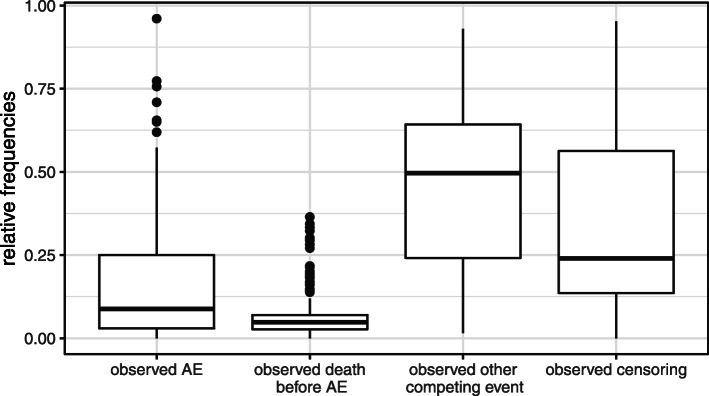
Relative frequencies of observed events. Number of patients with a specific type of event—either “observed AE”, “observed death before AE”, “observed other CE,” or “observed censoring”—divided by the total number of patients and for the maximal follow-up time

Figure 1 displays for the 186 types of AEs boxplots of the observed relative frequencies, i.e., the number of patients with a specific type of event divided by the total number of patients, namely of “observed AE,” “observed death before AE,” “observed other CE,” and “observed censoring” for the maximal follow-up time.

The figure illustrates a smaller amount of observed censoring compared to observed other, i.e., non-death CEs. That is, AE recording often ended due to death or other CEs such as treatment discontinuation preventing censoring of the time to AE. There are also much less death events than other CEs.

### Comparison of AE probability estimators

**Fig. 2:**
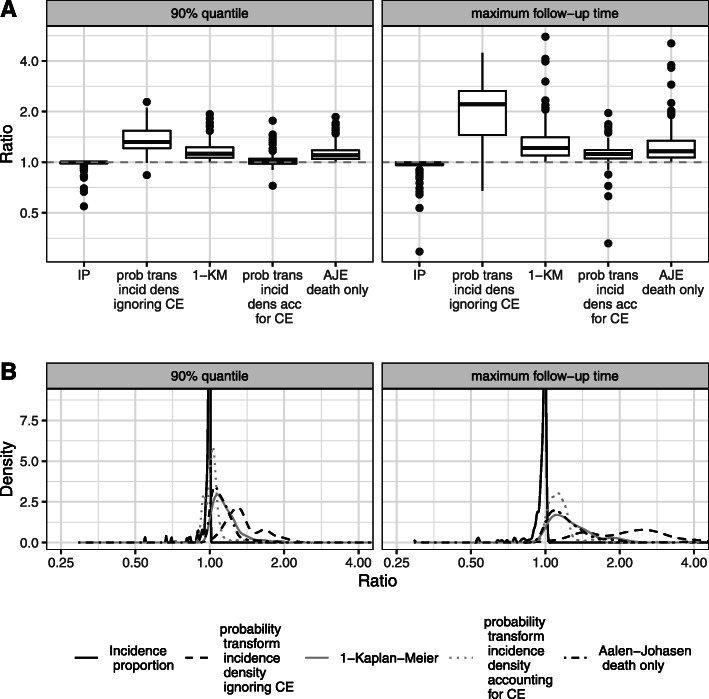
Ratios of one-sample estimators divided by gold-standard AJE. **A** Accepting the all event definition of CEs as gold-standard, the ratios of one-sample estimator divided by gold-standard AJE are displayed. Two different evaluation times are displayed. The left boxplots are the results for the estimators being evaluated at the 90% quantile and the right boxplots are the results of the evaluation time with no restriction, i.e., at the end of follow-up. The following abbreviations are used for the estimators: incidence proportion (IP), probability transform of the incidence density ignoring CE (prob trans incid dens ignoring CE), one minus Kaplan-Meier (1-KM), probability transform of the incidence density accounting for CE (prob trans incid dens acc for CE), death only AJE (AJE death only). **B** Plots of the kernel density estimates of the ratios of the AE probability of the estimators divided by the gold-standard AJE

Panel A of Fig. 2 shows box plots of the ratio of the one-sample estimators defined earlier divided by the gold-standard AJE for the maximum follow-up time and one earlier evaluation time chosen as to the 90% quantile. As the incidence proportion implicitly accounts for CEs (but not for censoring) as explained above, the small amount of censoring which is a consequence of the high amount of other CEs explains why the incidence proportion and the AJE are of similar size in many situations. But it has to be emphasized that in extreme cases an underestimation of up to 70% was present.

Both one minus Kaplan-Meier and the probability transform incidence density ignoring CE overestimate the AE probability, and this is also true for the AJE that only considers death before AE as competing. Interestingly, the probability transform incidence density ignoring CE appears to be worst, while the probability transform incidence density accounting for CE performs much better than the other three procedures which are clearly biased resulting in extreme overestimation in many situations, up to a factor of five. These biases become less pronounced when looking at earlier evaluation times which prevent CEs and censoring after the respective end of evaluation time to enter calculations.

### Impact on frequency categories

The impact on frequency categories is illustrated in Table 2, where we have exemplarily chosen the maximum follow-up time as most representative exposure period.

**Table 2:** The impact of the choice of one-sample estimator on AE frequency categories for the maximal follow-up time

		Very rare	Rare	Uncommon	Common	Very common
(a) Gold-standard AJE						
Incidence	Very rare	**6**				
proportion	Rare		**0**			
	Uncommon			**6**		
	Common				**86**	2
	Very common					**86**
Probability	Very rare	**6**				
transform	Rare		**0**			
incidence	Uncommon			**3**		
density	Common			3	**51**	
ignoring CE	Very common				35	**88**
1-Kaplan-	Very rare	**6**				
Meier	Rare		**0**			
	Uncommon			**4**		
	Common			2	**72**	
	Very common				14	**88**
Probability	Very rare	**6**				
transform	Rare		**0**			
incidence	Uncommon			**4**		
density	Common			2	**79**	1
accounting	Very common				7	**87**
for CE						
Aalen-	Very rare	**6**				
Johansen	Rare		**0**			
death only	Uncommon			**5**		
	Common			1	**73**	
	Very common				13	**88**
(b) AJE death only						
Incidence	Very rare	**6**				
proportion	Rare		**0**			
	Uncommon			**5**	1	
	Common				**73**	15
	Very common					**86**

Deviations from the AJE are the non-diagonal entries. The first rows consider the gold-standard AJE and the last five rows the comparison of the incidence proportion and the AJE (death only) estimator. Diagonal entries are set in bold face. Non-diagonal zeros are omitted from the display

Some switches to neighboring categories are detected. The probability transform of the incidence density ignoring CEs derives a higher AE frequency category for 38 types of AEs, and the one minus Kaplan-Meier estimator for 16 types of AEs. The probability transform of the incidence density accounting for CE obtains a higher category for nine types of AEs but also a lower category for one type of AE. Here, the definition of the CE is again of importance. The death only AJE categorizes 14 types of AEs to a higher category than the gold-standard AJE. The incidence proportion derives only two times a different AE frequency category than the gold-standard AJE. The good performance of the incidence proportion is closely connected to the CE definition, i.e., the maturity of data at the time of the analysis. If in the comparison to the incidence proportion the AJE (death only) is used instead of the gold-standard, the category common instead of very common is obtained for 15 types of AEs and one type of AE is categorized to uncommon using the incidence proportion but to common using the AJE that only considers death as a CE estimator (see last five rows of Table 2).

### Random effects meta-analysis

In a meta-analysis of the log-ratio of the incidence proportion divided by the AJE evaluated at the maximum follow-up time, the average ratio was found to be 0.972 with a 95% confidence interval of [0.965,0.980]. The respective result for the probability transform incidence density ignoring CE was 2.097 [1.994,2.205] and for one minus Kaplan-Meier was 1.214 [1.184,1.245]. Accounting for competing risks in an incidence densities-analysis (probability transform incidence density accounting for CE) gave a result of 1.130 [1.112,1.150], while the AJE (death only) estimator lead to an average of 1.170 [1.145,1.195]. These results confirm the visual impression gathered from the boxplots in panel A of Fig. 2, but we note that panel A of Fig. 2 also displays biases in individual trials which are much larger than the meta-analytical averages.

### Random effects meta-regression

The influence of different factors on the size of the bias was investigated in univariable and multivariable meta-regression. The percentage of censoring, the size of the AE probability estimated by the gold-standard AJE, and the evaluation time point were considered and included as covariates in the meta-regression models. In Table 3, results are exemplarily displayed when evaluating estimators using the maximum follow-up time as evaluation time.

**Table 3:** Univariable and multivariable meta-regression

			Probability transform		Probability transform	
		Incidence	incidence density	1-Kaplan-Meier	incidence density	AJE
		proportion	ignoring CE		accounting for CE	death only
**Univariable meta-regression**						
% censoring	Average risk ratio	0.974 [0.964; 0.983]	2.308 [2.217; 2.403]	1.257 [1.226; 1.288]	1.101 [1.086; 1.116]	1.201 [1.175; 1.228]
	10% increase	0.999 [0.996; 1.002]	0.916 [0.903; 0.929]	0.973 [0.965; 0.980]	1.026 [1.021; 1.031]	0.979 [0.972; 0.986]
%CEs	Average risk ratio	0.976 [0.969; 0.984]	2.191 [2.141; 2.243]	1.240 [1.214; 1.267]	1.124 [1.109; 1.140]	1.190 [1.168; 1.213]
	10% increase	1.003 [1.000; 1.006]	1.127 [1.117; 1.138]	1.036 [1.028; 1.045]	0.977 [0.971; 0.982]	1.029 [1.021; 1.036]
Size of AE	Average risk ratio	0.973 [0.966; 0.980]	2.105 [2.005; 2.210]	1.215 [1.185; 1.246]	1.131 [1.112; 1.150]	1.171 [1.146; 1.197]
probability	increase of 0.1	0.996 [0.992; 1.000]	0.954 [0.930; 0.980]	0.995 [0.982; 1.008]	0.993 [0.984; 1.003]	0.993 [0.982; 1.004]
Evaluation	Average risk ratio	0.972 [0.964; 0.980]	2.094 [1.994; 2.199]	1.214 [1.184; 1.244]	1.131 [1.112; 1.150]	1.170 [1.145; 1.195]
time	one additional year	0.993 [0.987; 1.000]	1.054 [1.021; 1.087]	1.015 [0.999; 1.033]	0.996 [0.986; 1.007]	1.013 [0.998; 1.027]
**Multivariable meta-regression**						
Average risk ratio		0.976 [0.966; 0.985]	2.407 [2.348; 2.468]	1.277 [1.246; 1.308]	1.097 [1.082; 1.113]	1.218 [1.192; 1.245]
%censoring 10% increase		0.997 [0.994; 1.000]	0.890 [0.882; 0.899]	0.965 [0.957; 0.973]	1.028 [1.023; 1.034]	0.972 [0.965; 0.979]
Size of AE probability increase of 0.1		0.995 [0.991; 0.999]	0.893 [0.882; 0.904]	0.972 [0.961; 0.983]	1.008 [1.000; 1.016]	0.975 [0.965; 0.985]
Evaluation time one additional year		0.994 [0.988; 1.000]	1.036 [1.021; 1.051]	1.014 [1.000; 1.027]	1.003 [0.995; 1.011]	1.011 [0.999; 1.024]

Average risk ratio and multiplicative change by 10% increase in censoring, 10% increase in CEs, one additional year of observation or a 0.1 greater AE probabiltiy. Thereby, the size of the AE probability is estimated by the gold-standard AJE

Covariates were centered, i.e., the row “average risk ratio” contains the average ratio of the estimator of interest and the AJE if the covariate takes its mean. Those means were 31.5% censoring, 52.6% CEs, 971 days maximum follow-up time, and a size of the AE probability estimated by the AJE of 0.165. For example, for the comparison of the incidence proportion and the AJE, the estimated average ratio of the two estimators in a trial with 31.5% censoring is 0.974. Furthermore, in a trial with 10% more censoring the estimated average ratio is increased by the factor 0.999 but the unit value is contained in the corresponding confidence interval. So, the amount of underestimation by the incidence proportion which does not account for censoring slightly increases with an increasing amount of censoring. Considering the estimators that either do not (probability transform incidence density ignoring CE, one minus Kaplan-Meier) or only partially (AJE (death only)) account for CE, one finds that both a higher amount of censoring and a higher AE probability decrease the amount of overestimation. The explanation goes hand in hand with the increased average ratios for higher amounts of CEs as these estimators do account for censoring, and increased censoring will, in general, lead to a smaller amount of observed CEs. Likewise, a higher AE probability will, in general, lead to a smaller probability of CEs.

These results are confirmed by the multivariable meta-regression. The amount of CEs is not included in the multivariable meta-regression as there is a strong dependence with the amount of censoring and the size of the AE probability estimated by the gold-standard AJE.

### Variability

Even though on average the incidence proportion does well in this sample of selected AEs, the possible variability must not be neglected.

Considering the plots of the kernel density estimates of the ratios of the different estimators of the AE probability in panel B of Fig. 2, the ratio of incidence proportion and the gold standard is most often close to one. But there are also peaks of the estimated kernel density at smaller ratios indicating that the estimators are not always comparable. For the ratio of the probability transform of the incidence density accounting for CEs and the gold standard most values are slightly larger than one at the maximum follow-up time. At the earlier follow-up time according to the 90% quantile, the peak is closer to one with less variability present. The ratios of the one minus Kaplan-Meier and death only AJE to the gold standard have few values close to one. For the majority of AE types, these two estimators largely overestimate the AE probability. Both plots illustrate pronounced variability for probability transform of the incidence density ignoring CE.

### Exemplary results from single trials

A closer look is taken at single AE types in trials for which extreme under- or overestimation is present, i.e., extreme values in the right panel boxplots in Fig. 2. For example, the largest underestimation of the incidence proportion is for an AE which is only observed for three out of 274 patients. This corresponds to an incidence proportion of 0.011. However, an AJE estimate of 0.037 is obtained. This corresponds to a ratio of 0.294 with a 95% confidence interval of [0.084; 1.025], where the confidence interval has been obtained using the bootstrap. As 27.0% of the observations for this type of AE are censored, the amount of censoring is below the mean censoring rate of all types of AEs. Moreover, for this type of AE, 17 deaths (6.2%) and 180 other CEs (65.7%) are observed. This type of AE does not only contribute the largest underestimation of the incidence proportion but also of the probability density of the incidence density accounting for CEs for which an estimate of 0.012 is obtained (ratio of 0.329 with 95% CI [0.094; 1.148]). Furthermore, for this type of AE, the largest overestimation of the one minus Kaplan-Meier estimator (estimate of 0.208 and ratio of 5.575 [1.813; 17.147]) and the AJE (death only) (estimate of 0.190 and ratio of 5.090 [1.815; 14.276]) is calculated. These impressive ratios are partly due to the small value of the gold-standard AJE estimate, but we stress that also the difference between one minus Kaplan-Meier and the gold standard is quite pronounced (0.208 vs. 0.037).

In another extreme example with a higher AE probability, the obtained incidence proportion is 0.059 and the AJE estimate is 0.109 (ratio 0.534 [0.529; 0.540]). For this type of AE, many censored observations are present (63.3% of 752 patients). Moreover, 44 AEs are observed, 137 deaths (18.2%), and 95 other CEs (12.6%). Here, due to the high amount of censoring, one can expect in advance the incidence proportion not doing well.

### Role of censoring

**Fig. 3:**
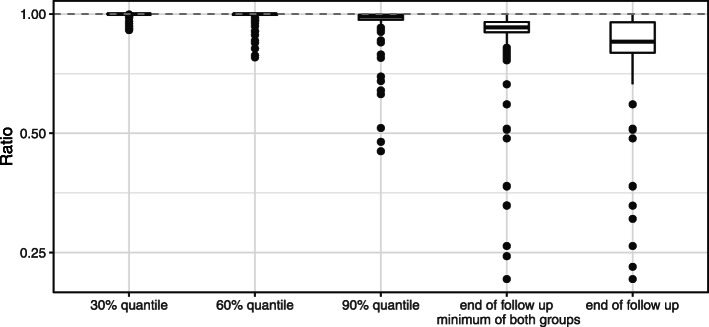
Ratios for the composite endpoint. Ratios of incidence proportion of the composite endpoint combining AE and CE divided by composite one minus Kaplan-Meier estimator

To explicitly investigate the role of censoring without the methodological complication of CEs, the composite endpoint combining AEs and CEs is considered, which results in a single endpoint survival setting. As a consequence the gold standard in this setting is the one minus Kaplan-Meier estimator which is compared to the incidence proportion (see Fig. 3).

In the composite endpoint analysis, the underestimation of the incidence proportion is more pronounced than in the analyses of the AE probability presented above. One reason is that even in the presence of censoring for the one minus Kaplan-Meier estimator the type of the last event is most important. If the last event is an AE or CE, the one minus Kaplan-Meier estimator is equal to one, even though censoring has been observed at earlier follow-up times. The incidence proportion is only equal to one if no censoring is observed.

## Discussion

The starting point of the present investigation was that AE analyses in terms of AE probabilities, an important aspect of drug safety evaluations, should account for the time under observation and censoring if the latter is imposed by the data at hand. As an additional complication, the occurrence of AE (of a certain type) usually is subject to CEs such as death before AE. Survival analyses accounting for CEs is methodologically well established, but practical use lacks behind [25, 26]. Failure to account for censoring (e.g., incidence proportion) or CEs (e.g., one minus Kaplan-Meier) will generally lead to biased quantification of absolute AE risk. As outlined earlier, we therefore recommend using AJE as the non-parametric, unbiased estimator in the presence of both CEs and censoring. However, the amount of empirical bias with respect to the gold-standard has been unclear.

In this study, we confirmed that one minus Kaplan-Meier should not be used to estimate the cumulative AE probability, as it is bound to overestimate as a consequence of ignoring CEs. Interestingly, we found that the incidence proportion performed surprisingly well when compared to the gold-standard AJE. This does not imply that we recommend using the simpler incidence proportion as a reasonable alternative to the gold-standard. One reason for the observed performance of the incidence proportion may be a high amount of CEs before possible censoring. But not only the proportion of censoring but also the timing of the censoring are relevant as the first example of the single trials described in detail showed. This example led to the largest bias although the proportion of censoring was below average. The observed proportion and timing of censoring in this project are a consequence of twelve out of 17 trials being from oncology in, which compared to other therapeutic areas, AEs and CEs are often observed early during follow-up and censoring occurs much later. We also note that the observed constellation of CEs and censoring results from a sample of completed trials after the final analysis had been performed. The proportion of censoring may be different at the time point of a safety interim analysis of trials which are typically presented to data safety monitoring boards. For this situation, the different estimators may behave differently [27], and this reinforces our recommendation to use the AJE.

Therefore, this finding must not be interpreted as a carte blanche to use AE incidence proportions based on censored data. In fact, comparable performance of incidence proportion and AJE did not only rely on a high amount of CEs, but in particular on a careful definition of what kind of events constitute a CE as outlined earlier. In other words, use of the incidence proportion *implicitly* assumes events to be competing as defined in the “Methods” section. This aspect is somewhat subtle, but nicely highlighted by the fact that an analysis accounting for both censoring and only death as CEs (AJE (death only)) also led to overestimating AE risk, although the bias was not as pronounced as for one minus Kaplan-Meier. We consequently recommend careful a priori considerations of what events constitute CEs, guided by our operationalization given earlier. This informs both what the incidence proportion estimates (should there be no additional censoring) and which events should be handled as CEs using AJE.

We also found that previous worries about the constant hazard assumption underlying incidence densities were justified in that a simple transformation of the AE incidence density onto probabilities (probability transform incidence density ignoring CE) performed worst. However, accounting for CEs in an analysis that parametrically mimicked the non-parametric AJE performed better than both one minus Kaplan-Meier and AJE (death only); in this sense, ignoring CEs appeared to be worse than assuming constant hazards in our empirical study. We do not recommend using incidence densities accounting for CEs, because the AJE readily presents itself as a non-parametric alternative. However, if an analysis based on incidence densities is considered, we strongly recommend to incorporate incidence densities of CEs as detailed above. We also prefer the latter analysis over the Kaplan-Meier approach.

Most of the results were shown for the situation where the maximum follow-up time was chosen as evaluation time. When looking at earlier evaluation times defined by quantiles of the observed times, the resulting bias was, in general, less pronounced, due to a reduced relative frequency of CEs and of censoring (see Fig. 1). We regarded the situation of including all data up to the maximum follow-up time as the most relevant as this is the usual practice and also what is implicitly done by using the incidence proportion.

Our empirical study does have shortcomings. Using an opportunistic sample of randomized clinical trials from several sponsor companies, we have been able to illustrate possible consequences when quantifying AE risk in a manner that ignores censoring or CEs. However, being opportunistic, the sample does not lend itself to straightforward generalizations. More than two thirds of the trials were from oncology. These came with a high amount of CEs, which, in turn, led to comparable performances of incidence proportion and AJE. The vast majority of AEs were classified as “common” or “very common,” and AEs were also heterogeneous, coming from different therapeutic areas and were not necessarily treatment-related. These shortcomings were to be anticipated from an opportunistic sample, but it was our aim in this “real-world” setting to investigate and demonstrate which biases can occur in practice. These shortcomings do also impact the comparison of AE risks between treatment groups [21]. The observed results motivate future empirical investigations on how to quantify AE risk with the aim of better generalizability. As a further point, it was not the aim of this investigation to accurately estimate AE probabilities, but to compare different estimators. Our present study does not allow for a meaningful comparison of results in different diseases. Follow-up investigations concentrating on trials in specific disease areas are planned.

A methodological restriction is that we have focused our investigation on an analysis which mostly does not consider AEs after treatment discontinuation due to, e.g., disease progression in oncology. This restriction is, in particular, due to trial design when treatment discontinuation leads to stopping AE recording after a prespecified time period. In addition, in oncology, it is not uncommon that patients enter a different clinical trial after progression which further complicates matters. However, follow-up beyond treatment discontinuation is required to estimate a treatment policy estimand. In some settings such as health technology assessments, this is considered to be the estimand of primary interest [8]. The results of our investigation remain valid when including AE data after treatment discontinuation. In this case, other disease-related events leading to a stop of AE recording have to be considered as CEs, as e.g., death without prior AE.

Another methodological restriction is that we did not consider recurrent AEs, but only first events. It is desirable to consider more complex event histories, also beyond time-to-first-event. However, any such consideration will need to account for CEs (and censoring), and our investigation therefore also informs methodological considerations for analyzing such more complex event histories. In other words, both AEs after treatment discontinuation and recurrent AEs will still be subject to CEs.

In a forthcoming follow-up paper on comparing groups [21], we will use the data of the same trials to compare the same estimators as in this paper in terms of the relative risk, quantified through the risk ratio at the shorter of the follow-up times in the two groups. Furthermore, we will look at hazard-based estimators as the ratio of incidence densities and the hazard ratio from Cox regression. Again, we will argue why the AJE is also the most suitable estimator for group comparisons in terms of the relative risk and why it is crucial to consider the hazard ratio.

As we find in these two papers, commonly used methods such as incidence proportions, incidence densities, or Kaplan-Meier are all biased and therefore inappropriate to quantify AE risk in the presence of varying follow-up times, CEs and censoring. It is important to note that this bias is a statistical property of any of these estimators and *independent* of the purpose we use any of these estimators for, i.e., whether we quantify the risk for a prespecified or emerging AE, or estimate AE risk in a given therapeutic area, or want to detect a different AE signal between two treatment arms. Replacing existing estimators, primarily the incidence proportion, by the AJE would require definition of CEs upfront, but that appears feasible as CEs can typically be defined on a trial level and then equally be applied to any quantification of AE risk in that specific trial. We thus invite consideration whether existing guidelines should be updated advocating AJE.

Key guidelines for development and reporting of RCTs are those issued by the International Council for Harmonization (ICH). Methods to analyze safety data is touched upon in several of these, e.g., E2, E3, or E9. They are all describing analysis methods, primarily incidence proportion and incidence density.

ICH E2E [7] talks about “Identified risks that require further evaluation” and requires reporting of “frequencies” for these. We argue that in fact what is of interest here is the AE risk as defined above and to properly quantify these we recommend the AJE. Similarly, ICH E3 [28] requires tabulating “rate of occurrence” or “event rates,” again without being specific about what precisely is meant by that. Furthermore, it is mentioned that “Under certain circumstances, life table or similar analyses may be more informative than reporting of crude adverse event rates.” We interpret this as estimates of survival functions in a time-to-first-event analysis are to be provided, in order to estimate AE risk. Then again, we would recommend AJE for that purpose.

Also the key efficacy guideline, E9 [29], has a section on safety. ICH E9 explicitly asks for “...appropriate use of survival analysis methods to exploit the potential relationship of the incidence of adverse events to duration of exposure and/or follow-up,” so accounting for varying follow-up. In addition, “The risks associated with identified adverse effects should be appropriately quantified to allow a proper assessment of the risk/benefit relationship.” We read this as need for proper quantification of AE risk, and as we have shown, this is only possible by properly accounting for CEs and using AJE.

As discussed above, the EMA’s anticancer guideline [17] states “...Kaplan-Meier analysis of selected AEs, which considers censoring of events, may be useful.” but without being specific about which “events” to censor. This again asks for proper quantification of AE risk but suggests a potentially biased method.

Finally, an extension of the CONSORT statement on reporting harms [18] also recommends “Kaplan–Meier curves showing cumulative incidence of important adverse events can be helpful,” but neither discusses censoring nor CEs.

## Conclusion

Our recommendation is to “play it safe” and to use the AJE whenever the risk for AEs is to be quantified in a time-to-first-event analysis and neither hope for a small amount nor a large amount of CEs nor a favorable interplay of the distributions of the times of AEs, CEs, and censorings. In the former case, one minus Kaplan-Meier might work well, while in the latter two cases the incidence proportion might do so. We recommend using the AJE which equals one minus Kaplan-Meier in the absence of CEs and equals the incidence proportion in the absence of censoring and does allow for presence of both CEs and censoring. Future revisions of guidelines for reporting AEs should, therefore, consider advocating the AJE instead of incidence proportion, incidence density, and one minus Kaplan-Meier.

## Data Availability

Individual trial data analyses were run within the sponsor organizations using SAS and R software provided by the academic project group members. Only aggregated data necessary for meta-analyses were shared and meta-analyses were run centrally at the academic institutions. A markdown file providing exemplary code to compute all the estimators discussed in this paper for a given dataset is available on github: https://github.com/numbersman77/AEprobs. The corresponding output is available as html file: https://numbersman77.github.io/AEprobs/SAVVY_AEprobs.html.

## Abbreviations

AE: Adverse event
CE: Competing event
SAVVY: Survival analysis for AdVerse events with VarYing follow-up times
